# Media Multitasking and Cognitive Abilities: A Multi-Domain Assessment of Attention, Working Memory, Convergent Associative Thinking, and Fluid Reasoning

**DOI:** 10.3390/jintelligence14080175

**Published:** 2026-08-01

**Authors:** Tindara Caprì

**Affiliations:** Department of Human Sciences, Link Campus University, Via del Casale di S. Pio V, 44, 00165 Rome, Italy; t.capri@unilink.it

**Keywords:** media multitasking, attention, working memory, creativity, fluid intelligence, gender

## Abstract

The present study addressed a timely and important issue in cognitive and media psychology about the relationship between media multitasking and cognition. This study examined attention control, working memory, convergent associative thinking, and fluid reasoning, examining multiple functions within the same analytical framework and moving beyond the narrow focus on single cognitive domains that characterizes much prior research. The main aim was to deepen the understanding of the associations of media multitasking with these core domains of human cognition. A total of 214 healthy young adults (18–30 years) completed the Short Media Multitasking Questionnaire, Raven’s Progressive Matrices, the Digit Span Test, the Trail-Making Test, and the Remote Associates Task. Multivariate general linear models and subsequent regression analyses were performed while controlling for age and gender. The results did not reveal a significant overall association between media multitasking and the combined cognitive outcomes. At the univariate level, higher media multitasking scores were associated with lower performance on the Remote Associates Task, although the observed effect size was small. Given the cross-sectional design and reliance on self-reported media multitasking, longitudinal studies employing objective measures of media multitasking use are needed to clarify the robustness and directionality of these associations.

## 1. Introduction

Media multitasking can be defined as the simultaneous engagement with multiple media sources, such as texting while watching television, browsing social media while listening to music, or accessing online content during academic activities, has become increasingly prevalent (Luo et al., 2020; Minear et al., 2013; Ophir et al., 2009). It is now considered a normative behaviour within the digital age, facilitated by several factors including the ubiquitous availability of digital technologies, the seamless integration of media platforms, and the frequent exposure to multiple concurrent media stimuli across various contexts such as education, professional environments, and domestic settings (Schmidt et al., 2020).

Although media multitasking has been widely investigated during the last decade, the empirical evidence regarding its cognitive correlates remains inconsistent. Some studies have reported that frequent media multitasking is associated with poorer attentional control, reduced working memory capacity, and lower reasoning performance (Ophir et al., 2009; Minear et al., 2013), whereas other investigations have failed to replicate these findings or have reported only weak associations (Ji et al., 2026; May & Elder, 2018; Parry & le Roux, 2019; Qin et al., 2026; van der Schuur et al., 2015; Wannagat et al., 2024). This inconsistency may partly reflect important methodological differences across studies, including variability in participant characteristics, cognitive measures, and, importantly, the assessment of media multitasking itself.

Media multitasking has been operationalized using a variety of methodological approaches. Most studies have relied on self-report questionnaires, such as the Media Multitasking Index or its subsequent adaptations, which estimate habitual engagement in simultaneous media activities. Other investigations have employed ecological momentary assessment, media-use diaries, digital activity logs, or experimental multitasking paradigms to capture media behaviour under more naturalistic or controlled conditions. Because these approaches differ substantially in the aspects of behaviour they assess, methodological heterogeneity may contribute to the variability of findings reported in the literature. Consequently, comparisons across studies should consider not only differences in cognitive measures but also differences in the operationalization of media multitasking itself.

The present study addressed a timely and important issue in cognitive and media psychology about the relationship between media multitasking and cognition. Previous investigations have generally examined isolated cognitive domains, whereas the present study adopts a multidomain approach by simultaneously investigating attention, working memory (WM), convergent associative thinking, and fluid reasoning within the same analytical framework.

### 1.1. Media Multitasking and Cognitive Processing

Although these cognitive domains (attention, WM, convergent associative thinking and fluid reasoning) are partially interrelated through shared executive processes, they represent distinct constructs with different theoretical and psychometric foundations. WM primarily reflects the temporary maintenance and manipulation of information, fluid reasoning refers to the capacity to solve novel problems independently of acquired knowledge, and attentional control involves the efficient allocation of cognitive resources, whereas convergent associative thinking represents a specific component of creative cognition. Examining these domains simultaneously therefore allows investigation of whether media multitasking shows generalized or domain-specific associations with cognitive functioning.

From a theoretical point of view, Wickens’ Multiple Resource Theory (MRT) provides a framework for understanding how individuals allocate attention when performing multiple tasks simultaneously. In the context of media multitasking, this theory suggests that when engaging in media multitasking, individuals may allocate their attention across various media sources simultaneously, such as texting while watching TV or browsing the internet while listening to music. According to this theory (Wickens, 2008), the effectiveness of media multitasking depends on the compatibility between the characteristics of the tasks being performed and the available attentional resources. If multiple media sources require similar cognitive processes or compete for the same attentional resources (e.g., reading subtitles while listening to dialogue in a movie), performance may be compromised. Tasks that draw upon different attentional resources are more likely to be performed successfully concurrently, while tasks that compete for the same attentional resources may interfere with each other. For example, listening to instrumental music while reading may not interfere significantly because both tasks primarily involve different sensory modalities (auditory and visual) and cognitive processes. While attempting to engage in two tasks that require linguistic processing, such as writing an email while listening to someone speak, may lead to interference because both tasks require the allocation of attention to linguistic processing.

### 1.2. Media Multitasking, Attention Control and WM

Attention is a fundamental cognitive process crucial for information processing and task performance. Studies have shown that media multitasking is associated with reduced attentional control and increased distractibility. Continuous exposure to multiple media sources may lead to attentional fragmentation, hindering the ability to sustain focus on a single task. Additionally, frequent task-switching inherent in media multitasking may impair attentional resources, resulting in diminished cognitive performance. Furthermore, frequent switching between media sources may lead to increased cognitive load and reduced overall attentional capacity (Cain & Mitroff, 2011; Loh & Kanai, 2016; Ophir et al., 2009; Ralph et al., 2014; Uncapher & Wagner, 2018; Wiradhany et al., 2019).

Although many studies reported negative associations between media multitasking and attention, other research found no significant relationship between these variables (van der Schuur et al., 2015) or positive associations. For example, some studies found that individuals with a high level of media multitasking (HMM) perform better on multisensory integration tasks (Cain & Mitroff, 2011; Gorman & Green, 2016; Minear et al., 2013; Moisala et al., 2016; Ophir et al., 2009; Wiradhany et al., 2019).

These differential outcomes can be explained by the interplay of multiple cognitive functions, rather than single processes. This interplay includes how different functions like WM, attention, intelligence and others interact and influence each other to produce different results in individuals and across various contexts. Memory plays a crucial role in encoding, storing, and retrieving information, thus influencing various cognitive functions, such as attention. The constant influx of information from multiple media sources may overload WM capacity and cognitive resources, impairing encoding and consolidation processes. Some studies have confirmed this issue, showing that media multitasking is associated with worse WM performance, particularly in tasks requiring concurrent processing of visual and auditory information (Uncapher et al., 2016; Uncapher & Wagner, 2018). Moreover, frequent task-switching associated with media multitasking may disrupt memory retrieval, leading to retrieval inhibition and forgetfulness. However, the pattern of results produced on the association between media multitasking and WM is still less clear.

### 1.3. Media Multitasking, Convergent Associative Thinking and Fluid Reasoning

As stated above, cognitive functions are not isolated but rely on overlapping and complex networks of brain regions working together to enable cognitive processes. Consequently, other cognitive factors involved in human cognition may moderate and explain conflicting results, such as convergent associative thinking and fluid reasoning (Liu et al., 2022; Minear et al., 2013).

Convergent associative thinking plays a crucial role because media multitasking can either stimulate or hinder convergent associative thinking, depending on how it is utilized. On one hand, exposure to diverse media sources can provide inspiration and spark new ideas, but constant switching between tasks may disrupt the deep focus necessary for convergent associative thinking (Loh & Lim, 2020; Li et al., 2023). In addition, convergent associative thinking has received comparatively less attention within the media multitasking literature.

Research by Di Plinio et al. (2025) has indicated that fluid reasoning is managed by specific functional subcircuits, with higher abilities correlating to increased neural efficiency in fronto-parietal and sensorimotor networks. Fluid reasoning, which involves the ability to reason and solve novel problems independent of previously acquired knowledge, is also relevant because media multitasking could potentially impact fluid reasoning by either enhancing cognitive flexibility or overloading cognitive resources, thereby impairing problem-solving abilities.

There are still few and conflicting studies that have examined the relationship between media multitasking, convergent associative thinking, and fluid reasoning. For example, some research suggested a negative correlation between the frequency of media multitasking and fluid reasoning (Cain & Mitroff, 2011; Gorman & Green, 2016; Moisala et al., 2016; Ophir et al., 2009; Wiradhany & Nieuwenstein, 2017). Recent studies showed a positive correlation between media multitasking and fluid reasoning (Loh & Lim, 2020; Seddon et al., 2021).

To date, most studies have investigated individual cognitive domains separately, making direct comparisons difficult because different samples, assessment procedures, and statistical approaches have been employed. The present study extends previous research by examining several objectively assessed cognitive domains within the same sample using a common multivariate analytical framework while controlling for relevant demographic variables. This design allows a more comprehensive evaluation of whether habitual media multitasking is associated with broad cognitive functioning or with specific cognitive domains.

### 1.4. Research Aims, Research Significance, Research Questions (RQs)

Given contrasting findings yielded by the extant literature, not enough evidence exists to allow for the creation of convincing theoretical assumptions on the specific nature of the association of media multitasking with attention, WM, convergent associative thinking, and fluid reasoning. One of the key factors characterising conflicting outcomes can be the approach to measuring these associations. Most previous studies used self-report tools and measured these associations considering each variable singularly and separately; however, the relationship between media multitasking and cognitive processing is too complex to be explained by referring to a single cognitive process rather than the set of cognitive processes primarily involved during media multitasking behaviours.

In addition, contrasting findings can also be due to gender differences in the use of media and individual differences in everyday media multitasking. Regarding gender differences, although it is known that women engage more frequently in multitasking than men (Garaus & Wolfsteiner, 2023), prior studies have not extensively investigated the multiple associations between high and low media multitasking and cognitive functions while taking gender into account.

Individual differences in everyday media multitasking are typically measured using the Media Multitasking Index (MMI) of Ophir et al. (2009); this index allows us to examine and predict the cognitive and personality characteristics that are associated with media multitasking and identify those characteristics that can distinguish heavy media multitaskers (HMMs) from light media multitaskers (LMMs). By this measurement, research suggested that HMMs showed reduced performance in some cognitive domains compared to LMMs.

The present study aimed to deepen the understanding of the associations of media multitasking with attention, WM, convergent associative thinking and fluid reasoning. This research was significant as it addressed a gap in the literature by measuring and analyzing multiple cognitive domains, taking them all into account. By examining both cognitive processing factors and individual differences in media multitasking, this study provided a more comprehensive understanding of the relationship between media multitasking and cognitive functioning.

This study also aimed to address two research questions (RQs):

RQ1. Overall, what is the association between media multitasking, attention, WM, convergent associative thinking and fluid reasoning?
RQ2. More in depth, what is the association between the levels of media multitasking (high vs. low), attention, WM, convergent associative thinking and fluid reasoning?

Based on the available literature, we hypothesized that higher levels of habitual media multitasking would be associated with poorer performance across several cognitive domains. However, given the inconsistency of previous findings, the study was primarily designed to determine whether these associations would emerge uniformly across domains or whether they would be domain-specific.

## 2. Materials and Methods

### 2.1. Participants

A total of 214 healthy adults (108 females and 106 males) voluntarily participated in this study. The age range of participants was between 18 and 30 years (M = 22.77, SD = 3.56). The sample was recruited from the Link Campus University, and it was composed of students. Table 1 shows the demographic characteristics of the participants.

Restricting the sample to healthy young adults with a relatively homogeneous educational background reduced variability attributable to developmental and educational factors, thereby allowing for a more focused investigation of the associations between habitual media multitasking and cognitive functioning. However, this sampling strategy necessarily limits the generalizability of the findings, an issue that is addressed in the Section 4.1.

Prior to participation, all participants completed a structured clinical screening interview, conducted by a psychologist, during which inclusion and exclusion criteria were verified. Individuals reporting neurological disorders, psychiatric conditions, diagnosed Internet addiction, sleep disorders, current psychoactive medication, or substance abuse were excluded from participation. In addition, participants were asked demographic and psychiatric questions to gather information about their health condition, socioeconomic status and hours of device use to determine their eligibility for the study.

Inclusion criteria: 18–30 years of age, Italian nationality, no history of psychological or psychiatric disorder or illness disorders or head trauma, no history of learning disabilities, no history of intellectual or neurological disabilities, no substance use, no sleep disorders, no attention deficit hyperactivity disorder.

Exclusion criteria: current pharmacological treatments, smoking cannabis or taking drugs, gamer pro, Internet Addiction. Informed consent was obtained from all subjects involved in the study.

### 2.2. Instruments

Media Multitasking. The short version of the Media Multitasking Questionnaire (MMQ-S; Baumgartner et al., 2017) was used to measure the level of habitual media multitasking activities. We adapted this questionnaire into the Italian language. To ensure translation accuracy, the Italian version used in this study was obtained through forward translation by two native Italian-speaking researchers proficient in English (original version of the questionnaire), followed by consensus revision among bilingual researchers to ensure semantic equivalence with the original instrument. Internal consistency in the present sample was satisfactory for both the MMI and the subscales (Cronbach’s α = 0.80).

In the first section of the MMQ-S, participants reported the number of hours per week they usually spend doing each of nine activities. In the second section, participants indicated on a four-point scale how frequently they engage in the specific media activity simultaneously with each of the other eight activities. The response categories were: 1 (=never), 2 (=sometimes), 3 (=often), and 4 (=very often).

The nine media activities were: (1) watching TV, (2) reading, (3) listening to music, (4) talking on the phone, (5) sending messages via phone or computer (e.g., text messages, Ping, WhatsApp, Instant messaging), (6) using social network sites (e.g., Facebook, Twitter), (7) watching movies on the computer, (8) other computer activities (e.g., surfing on the web, photoshop, etc.), (9) playing video games.

This short version contains nine items selected from the original MMQ. The MMQ-S was originally composed of 72 items subdivided into nine media multitasking subscales. To increase the utility of the scale, the authors (Baumgartner et al., 2017) reduced the initial 72 items to 9 items. The most prevalent subscales were media multitasking with TV, sending messages, listening to music, and social network sites. Because listening to music is mainly a background activity that does not require full attention, Baumgartner et al. (2017) only categorised this as a secondary activity but not as a primary activity. Consequently, the final subscales were watching TV, sending messages, and social networking. Table 2 shows the final items.

The Media Multitasking Index (MMI) indicated the relative amount of media multitasking across different media categories. It was computed according to Ophir’s Formula (Ophir et al., 2009):

MMI = Σ^9^_i=1_ (m_i_ × h_i_)/h_total_

in which h_total_ is the total number of hours per week spent using all forms of media, mi denotes participants’ estimations of the frequency of use on other media activities while using a primary medium i, and hi refers to the number of hours per week spent using the primary medium i.

Convergent associative thinking. Convergent associative thinking was assessed using the Italian version of the Remote Associates Task (RAT; Mednick, 1962; Salvi et al., 2018). The task requires participants to identify a target word that is semantically associated with three seemingly unrelated cue words. Rather than measuring creativity as a broad construct, the RAT specifically assesses convergent associative processing, a component of creative cognition involving the integration of remote semantic associations. The RAT consists of 40 trials, each of which required the participant to find a convergent solution word, for example “MELON” associated with all three question words presented (e.g., “FRUIT”, “ROUND”, “GREEN”). The included words for each trial and the time available to solve each trial were presented for 20 s. If the participant could not locate the correct word within the set time limit, the word could no longer be transcribed afterward and was considered unresolved. Cronbach’s alpha (α) was 0.89, demonstrating high reliability for this measure (Salvi et al., 2018).

Fluid reasoning. Fluid reasoning was assessed using the abbreviated version of Raven’s Progressive Matrices (RSPM; Raven & Court, 1998), a standardized measure of nonverbal reasoning and abstract problem solving that is widely considered an indicator of fluid reasoning. This abridged version of the test, consisting of 36 items selected from sets B, C, and D, required participants to choose the correct figure from eight options in each item to complete the matrix presented. To get the correct answer, participants had to infer the underlying logic that linked the elements of the incomplete matrix to the possible options. Each correct answer received a score of 1, while no score was given for incorrect answers. The score obtained provided an indication of the subject’s Intelligence Quotient. The reliability of RSPM shows robust psychometric properties; internal consistency (Cronbach’s α) typically ranges between 0.80 and 0.90 for standard versions. Test–retest reliability often sits around 0.85.

WM. WM capacity was assessed using the Digit Span Test (Orsini et al., 1987). This task requires the temporary storage and manipulation of numerical information and represents one of the most extensively validated neuropsychological measures of verbal working memory. Participants are sequentially presented with a series of auditory figures, which must be reported immediately after listening to them, maintaining the order of presentation. The length of the sequence (span) starts with two digits and increases progressively during the activity, up to a maximum of nine digits, with a total of 14 tests to be completed. The participant advances to the next sequence only if they repeat the current sequence correctly. The activity continues until the participant fails two sequences of the same length. The score obtained corresponds to the maximum number of digits that the subject is able to repeat correctly in a sequence. The Digit Span Test shows moderate to high consistency. Internal consistency (Cronbach’s alpha) typically ranges from 0.70 to 0.80, while test–retest reliability ranges from 0.70 to 0.76 (Orsini et al., 1987).

Attention control. Attentional control, including processing speed, visuomotor scanning, sequencing, and set-shifting, was assessed using the Trail-Making Test (TMT; Giovagnoli et al., 1996). This task includes presenting circles containing a sequence of numbers (part A) or a sequence of numbers and letters (part B). In part A, participants had to connect the circles in sequential order as quickly as possible; on the other hand, in part B, they had to alternately link numbers and letters (1-A-2-B-3-C). Part A primarily evaluates visual scanning, psychomotor speed, and attentional processes, whereas Part B additionally requires cognitive flexibility, set shifting, and executive control. No time limit was set to complete the task. Completion time (in seconds) and number of errors for both Parts A and B were used as the outcome measures in accordance with standard neuropsychological practice. The TMT shows good to high test–retest reliability for both Part A and Part B, with coefficients generally falling in the r_s_ ≥ 0.50 range.

### 2.3. Procedure

Participants were tested individually in two sessions in a quiet room of an Italian University, from 9:00 a.m. to 12:00 p.m. This specific time frame is a standard protocol to ensure consistent arousal levels across participants. In the first session, participants performed the MMQ-S, while in the second session, cognitive tests were randomly administered.

### 2.4. Statistical Analysis

Statistical analyses were performed using IBM SPSS Statistics (Version 24.0) for Windows. Descriptive statistics of the dependent variables were tabulated and examined. Alpha level was set to 0.05 for all statistical tests. Associations between media multitasking and cognitive performance in multiple cognitive domains were examined using a multivariate general linear model with MMI as a continuous predictor, while controlling for age and gender. Significant multivariate effects were followed by separate multiple linear regression analyses for each cognitive outcome to estimate the unique association between MMI and performance in each cognitive domain.

Regression models are reported using unstandardized coefficients (B), standardized coefficients (β), standard errors, 95% confidence intervals, t statistics, *p* values, and coefficients of determination (R^2^). Effect sizes are reported as Cohen’s d for group comparisons where appropriate.

In addition to the primary continuous analyses, an exploratory comparison between heavy media multitaskers (HMMs) and light media multitaskers (LMMs) was conducted using the conventional ±1 SD classification adopted in the previous literature to select HMMs vs. LMMs, and two-sample *t*-tests were used to examine whether these participants differed in all variables considered in this study. This secondary analysis was included exclusively to facilitate comparison with earlier studies and should therefore be interpreted as exploratory.

Prior to inferential analyses, assumptions of normality, linearity, homoscedasticity, multicollinearity, and multivariate normality were examined. No substantial violations requiring alternative analytical procedures were observed.

## 3. Results

### 3.1. Descriptive Statistics and Correlation Matrix

Given the multi-domain and multivariate framing of this study, we reported descriptive statistics in Table 3 and bivariate associations for all main variables included in this study. Table 4 shows the bivariate correlations among the MMI, its categories, and the cognitive outcomes. Overall, the correlations between the MMI and cognitive measures were weak and largely non-significant. Specifically, the overall MMI was not significantly associated with WM, fluid reasoning, convergent associative thinking, or attention control. Among the media multitasking categories, only TV multitasking showed a small negative correlation with WM (r = −0.153, *p* < .05) and a small positive correlation with TMT Part A errors (r = 0.139, *p* < .05). As expected, the MMI was strongly correlated with its constituent categories (r = 0.728–0.858, *p* < .001), while the cognitive measures showed the expected pattern of intercorrelations (e.g., TMT Part A and Part B execution times: r = 0.667, *p* < .001). Overall, the correlation matrix indicates limited bivariate associations between media multitasking and cognitive performance, supporting the findings of the multivariate analyses.

### 3.2. Multivariate Analysis

The multivariate effect of media multitasking on the combined cognitive outcomes was not statistically significant (Wilks’ λ = 0.942, *F*(7, 2023) = 1.7971, *p* = 0.089, *n*^2^*_p_* = 0.15), indicating that media multitasking did not significantly predict overall cognitive functioning after controlling for demographic variables. In contrast, gender showed a significant multivariate effect (Wilks’ λ = 0.899, *F*(7, 2023) = 3.2478, *p* = 0.003, *n*^2^*_p_* = 0.56), whereas age was not significantly associated with the combined cognitive measures.

Subsequent univariate multiple regression analyses were performed for each cognitive outcome. Table 5 shows regression coefficients for the predictor (MMI) and covariates (age and gender), together with standard errors, confidence intervals, standardized coefficients, and significance values. Results indicated that higher media multitasking scores were associated with lower convergent associative thinking performance. No other significant associations emerged between media multitasking and the remaining cognitive domains. Overall, these results suggest that media multitasking was not associated with broad cognitive impairment; however, a small negative relationship with convergent associative thinking performance was observed independently of age and gender, but the effect size was small.

Secondly, we examined whether HMMs and LMMs differed in cognitive domains considered in this study. In MMI, there were statistically significant group differences *t*(98) = 15.17, *p* < .001, *d* = 0.85; however, the difference in MMI between the two groups is inherent to the grouping criterion and therefore cannot be interpreted as an empirical finding, but as a verification of the classification procedure.

### 3.3. Gender Differences in MMT Scores

Table 6 shows the means and standard deviation of men and women in media multitasking. Given the multivariate effect of gender, we verified whether there were significant differences between men and women in their MMT scores. A statistically significant group difference in MMI was found: *t*(98) = 2.81, *p* = .004, *d* = 0.56. Overall, women showed higher levels of media multitasking than men. More specifically, they reported higher levels of media multitasking when using social networks and messages, respectively, *t*(98) = 2.79, *p* = .001, *d* = 0.56; *t*(98) = 3.97, *p* < .001, *d* = 0.80.

As regards the comparison between HMMs and LMMs, there were group differences for the gender variable, *t*(98) = 3.45, *p* < .001, *d* = 0.78, confirming the multivariate effect of gender.

## 4. Discussion

The present study aimed to deepen the understanding of the associations between media multitasking and multiple cognitive domains, including selective attention, WM, convergent associative thinking, and fluid reasoning. Specifically, this research addressed a gap in the existing literature by simultaneously examining several cognitive functions within a multivariate framework rather than focusing on isolated domains. Contrary to our initial expectations, the findings provided limited evidence for generalized associations between media multitasking and cognitive functioning. Specifically, while higher levels of habitual media multitasking were modestly associated with lower performance on the Remote Associates Task, no statistically significant relationships emerged for fluid reasoning, working memory, or attentional performance after controlling for age and gender. These findings therefore only partially supported our initial hypothesis and suggest that any cognitive correlates of media multitasking may be domain-specific rather than generalized. More broadly, the present findings contribute to an ongoing debate that has produced inconsistent conclusions over the past decade. Early investigations frequently suggested that heavy media multitasking was associated with widespread deficits in attentional control and executive functioning, whereas more recent studies have generally reported weaker, inconsistent, or null associations. The findings appear to align more closely with this latter body of evidence, supporting the view that generalized cognitive disadvantages associated with habitual media multitasking may be less robust than initially proposed.

The multivariate analyses showed that media multitasking did not significantly predict the combined set of cognitive outcomes. This result partially contrasts with earlier studies reporting detrimental effects of heavy media multitasking on attentional control and executive functioning (Qin et al., 2026). At the same time, the present findings are consistent with more recent evidence suggesting that the relationship between media multitasking and cognition may be weaker and less generalized than initially proposed (Ji et al., 2026). By adopting a multivariate approach, the current study provides additional evidence that media multitasking may not uniformly impair cognitive functioning across domains.

The only statistically significant association observed concerned performance on the Remote Associates Task, with higher levels of multitasking predicting lower convergent associative thinking performance. Although previous literature has often linked multitasking behaviours to increased exposure to diverse information sources and potentially enhanced divergent thinking (Li et al., 2023), the present findings suggest that excessive concurrent media use may instead interfere with sustained cognitive engagement and flexible idea generation. One possible explanation is that continuous attentional switching and fragmented information processing may reduce the depth of cognitive elaboration required for creative performance. However, this finding should be interpreted cautiously. The observed effect size was small, the explained variance was limited, and statistical significance was close to the conventional threshold. Moreover, because several cognitive outcomes were examined simultaneously, this association should be regarded as preliminary and deserving of replication rather than definitive evidence of a robust relationship between habitual media multitasking and convergent associative thinking.

No significant association emerged between media multitasking and fluid reasoning. This finding is consistent with several recent investigations (Parry & le Roux, 2019; Ma et al., 2024; Wannagat et al., 2024) suggesting that higher-order reasoning abilities may be relatively resilient to habitual patterns of media use. Given that fluid reasoning reflects complex abstract problem-solving processes that rely on multiple interacting cognitive systems, it is plausible that media multitasking exerts, at most, only a limited influence on this domain.

Similarly, WM performance was not significantly associated with media multitasking. Although previous studies have reported conflicting findings, methodological differences—including participant characteristics, assessment procedures, and definitions of media multitasking—likely contribute substantially to the variability reported across the literature. Consequently, the present findings add to growing evidence suggesting that any relationship between media multitasking and WM may be considerably smaller than originally hypothesized.

The absence of significant associations on the TMT should also be interpreted with caution because performance reflects multiple cognitive processes—including attentional control, processing speed, visual scanning, and executive flexibility—rather than selective attention alone. Therefore, the present findings do not necessarily indicate that all attentional processes are unrelated to habitual media multitasking.

Gender differences were also observed in media multitasking behaviours. Female participants reported higher levels of overall media multitasking, as well as greater multitasking involving social media and sending messages, compared with male participants. These findings are in line with prior research showing that women tend to engage more frequently in communication-oriented and socially interactive media activities (Garaus & Wolfsteiner, 2023). Importantly, gender also demonstrated a significant multivariate effect on cognitive outcomes, supporting the importance of controlling for demographic variables when examining the cognitive correlates of media multitasking.

As regards methodological aspects, compared to previous studies, the present study examined the relationship between media multitasking, attention, convergent associative thinking, WM, and fluid reasoning, considering them simultaneously in the analysis, and it introduced two variables in the examination, such as WM and attention. Additionally, contrary to most previous studies, this study did not use self-reporting to measure these cognitive domains, but psychometric tests. These methodological choices constitute important contributions to the field of psychological and cognitive research, as they address methodological inconsistencies that contributed to conflicting findings in the literature on media multitasking and cognition.

### 4.1. Limitations

Some limitations should be acknowledged. First, the cross-sectional design precludes causal inferences regarding the directionality of the observed associations. Second, although cognitive outcomes were measured objectively, media multitasking was assessed solely via self-report measurement, which may be influenced by recall biases and subjective interpretation. In addition, each cognitive domain was assessed with a single measure; although the selected tests are widely used and appropriate, they capture specific operationalizations of attention, WM, convergent associative thinking, and fluid intelligence. Third, the sample mainly consisted of Italian young adults and students, limiting the generalizability of the findings to other age groups or other categories. Consequently, the findings should be interpreted within the context of young Italian university students and may not generalize to individuals with different educational backgrounds, age groups, occupational profiles, or cultural settings.

An important aspect of the present study concerns the magnitude of the observed effects. Across analyses, media multitasking accounted for only a small proportion of the variance in cognitive performance. Such modest effect sizes suggest that media multitasking represents only one of many factors potentially associated with individual differences in cognition. Cognitive performance is influenced by numerous biological, psychological, educational, motivational, and environmental determinants, making relatively small effects unsurprising within cross-sectional observational research.

Future research could benefit from using multiple indicators of convergent associative thinking, attention, WM, and fluid reasoning to test whether the present findings generalize across different operationalizations of these constructs. Future studies would benefit from combining standardized neuropsychological assessment with objective digital behavioural measures, ecological momentary assessment, passive smartphone sensing, or digital activity logs. In addition, although a formal cross-cultural validation study was beyond the scope of the present investigation, future studies should further evaluate the psychometric properties of the Italian version of the MMQ-S using larger and more heterogeneous samples. Furthermore, longitudinal and experimental designs will be essential to clarify whether media multitasking precedes cognitive differences or whether individuals with specific cognitive profiles preferentially adopt multitasking behaviours. In addition, the inclusion of multiple indicators for each cognitive domain would permit latent-variable modelling capable of distinguishing domain-general from domain-specific cognitive associations. Finally, future studies should include larger and more heterogeneous samples to evaluate the robustness and external validity of the present findings.

### 4.2. Practical Implications

The findings of the present study had several practical implications. It was found that women exhibited higher levels of media multitasking than men, so if gender influenced media multitasking behaviours, it could affect the generalizability of cognitive research findings. Studies that did not account for these differences might overlook important variables, leading to incomplete or biased conclusions.

Although the present findings do not support the existence of generalized cognitive differences associated with media multitasking among healthy young adults, they should not be interpreted as evidence that media multitasking is cognitively harmless in every context. Rather, the results suggest that concerns regarding widespread cognitive impairment associated with everyday media multitasking may require greater nuance. Educational and clinical recommendations should therefore continue to be guided by converging evidence from longitudinal, experimental, and ecologically valid studies rather than by findings from individual cross-sectional investigations. In educational and professional settings, where multitasking is often inevitable, knowing that it does not necessarily impair cognitive abilities could influence the design of work environments, teaching strategies, and productivity tools.

## 5. Conclusions

In conclusion, the present study contributes to the growing literature investigating the cognitive correlates of media multitasking by simultaneously examining multiple objectively assessed cognitive domains within a single analytical framework. The findings provide limited evidence for generalized cognitive differences associated with media multitasking among healthy young adults, while suggesting a modest association with convergent associative thinking that requires further replication. This study reveals no significant differences between high and low multitaskers. Gender, however, emerges as a significant predictor of media multitasking, with women reporting higher levels of media multitasking, particularly in relation to social networking and sending messages. These results highlight the complexity of the relationship between media multitasking and cognitive functioning and underscore the need for more integrative approaches in future research. Overall, the results support an increasingly nuanced interpretation of the media multitasking literature and highlight the importance of rigorous methodology, objective cognitive assessment, and longitudinal research in clarifying the relationship between media multitasking and cognition.

## Figures and Tables

**Table 1:** Demographic characteristics of participants.

Variables	Values
Gender	
Females, *n*	108
Males, *n*	106
Highest educational level	
High School, *n*	92
Bachelor’s degree, *n*	72
Master’s degree, *n*	50
Postgraduate degree, *n*	0
Socioeconomic status	
High, *n*	113
Middle, *n*	101
Low, *n*	0

**Table 2:** List of items of the short media multitasking questionnaire.

Items in the Short Media Multitasking Questionnaire
While watching TV, how frequently do you participate in the following activities: using social networking sites sending messages via computer or phone listening to music 2. While using social networking sites, how frequently do you participate in the following activities: watching TV listening to music sending messages via computer or phone 3. While sending messages via phone or computer, how frequently do you participate in the following activities: watching TV using social networking sites listening to music

**Table 3:** Means (M), standard deviations (SD), and observed ranges for all main variables.

Variables	M	SD	Minimum	Maximum
WM	6.37	0.85	4	9
Fluid reasoning	20.48	2.95	15	32
Convergent associative thinking	1.70	1.34	0	6
TMT Part A execution time	19.65	5.80	9.46	36.98
TMT Part B execution time	38.50	1.04	13.09	102.20
TMT Part A errors	0.21	0.90	0	5
TMT Part B errors	2.04	2.38	0	8
MMT tv	5.78	1.37	2.33	9.33
MMT social	6.26	1.54	2.33	9.33
MMT messages	5.84	1.39	2.33	9.33
MMI	13.98	2.67	8.11	20.44

**Table 4:** Correlation matrix including the MMI and the cognitive outcomes.

	WM	Fluid Reasoning	Convergent Associative Thinking	TMT Part A Execution Time	TMT Part B Execution Time	TMT Part A Errors	TMT Part B Errors	MMT tv	MMT Social	MMT Messages	MMI
WM	1	−0.040	0.190 **	−0.072	−0.120	0.054	0.060	−0.153 *	0.032	0.042	−0.053
Fluid reasoning	−0.040	1	0.027	−0.090	0.085	−0.017	0.032	−0.001	−0.019	−0.068	−0.023
Convergent associative thinking	0.190 **	0.027	1	−0.016	0.057	0.005	−0.086	−0.090	−0.093	−0.004	−0.100
TMT Part A execution time	−0.072	−0.090	−0.016	1	0.667 **	−0.132	−0.072	−0.068	−0.055	−0.090	−0.082
TMT Part b execution time	−0.120	0.085	0.057	0.667 **	1	0.172 *	−0.520 **	0.074	−0.027	−0.034	0.016
TMT Part A errors	0.054	−0.017	0.005	−0.132	0.172 *	1	−0.195 **	0.139 *	0.059	−0.037	0.099
TMT Part B errors	0.060	0.032	−0.086	−0.072	−0.520 **	−0.195 **	1	−0.030	0.069	−0.059	0.014
MMT tv	−0.153 *	−0.001	−0.090	−0.068	0.074	0.139 *	−0.030	1	0.304 **	0.275 **	0.737 **
MMT social	0.032	−0.019	−0.093	−0.055	−0.027	0.059	0.069	0.304 **	1	0.716 **	0.858 **
MMT messages	0.042	−0.068	−0.004	−0.090	−0.034	−0.037	−0.059	0.275 **	0.716 **	1	0.728 **
MMI	−0.053	−0.023	−0.100	−0.082	0.016	0.099	00.14	0.737 **	0.858 **	0.728 **	1

* *p* = .05; ** *p* < .001.

**Table 5:** Multiple regressions for cognitive domain, including MMI as a continuous predictor and age and gender as covariates.

Outcome	Predictor Covariates	β	B	SE	95% CI	*p*	R^2^
WM	MMI	−0.118	−0.038	0.023	−0.083, 0.007	0.097	0.047
	age	−0.056	−0.013	0.017	−0.047, 0.020	0.426	
	gender	0.200	0.341	0.122	0.101, 0.582	0.006	
Fluid reasoning	MMI	−0.032	−0.035	0.079	−0.191, 0.121	0.658	0.025
	age	−0.153	−0.127	0.059	−0.243, −0.011	0.033	
	gender	−0.082	−0.482	0.425	−1.321, 0.356	0.258	
Convergent associative thinking	MMI	−0.141	−0.071	0.036	−0.141, −0.000	0.049	0.041
	age	0.046	0.017	0.027	−0.035, 0.070	0.514	
	gender	0.185	0.496	0.192	0.117, 0.874	0.011	
TMT Part A execution time	MMI	−0.040	−0.087	0.156	−0.395, 0.220	0.575	0.024
	age	0.057	0.092	0.116	−0.136, 0.321	0.427	
	gender	−0.113	−1.305	0.836	−2.954, 0.344	0.120	
TMT Part B execution time	MMI	0.044	0.297	0.488	−0.665, 1.260	0.543	0.009
	age	0.010	0.051	0.363	−0.665, 0.767	0.888	
	gender	−0.095	−3.422	2.621	−8.589, 1.746	0.193	
TMT Part A errors	MMI	0.112	0.038	0.025	−0.010, 0.086	0.123	0.012
	age	0.045	0.011	0.018	−0.025, 0.047	0.530	
	gender	−0.013	−0.023	0.132	−0.282, 0.237	0.863	
TMT Part B errors	MMI	0.026	0.024	0.064	−0.103, 0.150	0.715	0.022
	age	−0.111	−0.075	0.048	−0.169, 0.020	0.120	
	gender	−0.128	−0.608	0.345	−1.288, 0.071	0.079	

**Table 6:** Means (M) and standard deviation (SD) of men and women in MMT scores.

MMQ-S	Men Group	Women Group	*t*	*p*	*d*
	M (SD)	M (SD)			
MMI	13.25 (2.40)	14.69 (2.74)	2.81	.004	0.56
MMT tv	5.64 (1.43)	5.91 (1.31)	1.61	.070	0.20
MMT social	5.84 (1.42)	6.66 (1.56)	2.79	.001	0.56
MMT messages	5.31 (1.18)	6.35 (1.41)	3.97	.001	0.80

## Data Availability

The data presented in this study are available on request from the corresponding author due to privacy reasons.
